# Immunological Changes in Monocyte Subsets and Their Association With Foxp3^+^ Regulatory T Cells in HIV-1-Infected Individuals With Syphilis: A Brief Research Report

**DOI:** 10.3389/fimmu.2019.00714

**Published:** 2019-04-09

**Authors:** Na Guo, Lifeng Liu, Xiaodong Yang, Ting Song, Guanxin Li, Li Li, Taiyi Jiang, Yanqing Gao, Tong Zhang, Bin Su, Hao Wu

**Affiliations:** ^1^Center for Infectious Diseases, Beijing Youan Hospital, Capital Medical University, Beijing, China; ^2^Beijing Key Laboratory for HIV/AIDS Research, Beijing, China

**Keywords:** syphilis, HIV-1, MSM, monocyte subsets, Treg cells

## Abstract

The incidence of syphilis has increased dramatically in men who have sex with men (MSM), especially those with HIV-1 infection. *Treponema pallidum* and HIV-1 are bidirectionally synergistic, accelerating disease progression reciprocally in co-infected individuals. We have shown that monocytes have different effects on T helper cells at different stages of HIV-1 infection. However, the immunological changes in the three monocyte subsets and in regulatory T cells (Tregs), and the associations between these cell types during syphilis infection among HIV-1-infected MSM remain unclear. Herein, we used cell staining methods to explore changes in monocyte subsets and Tregs and any associations between these cells. We found that the frequency of classical monocytes was higher in the rapid plasma reagin (RPR^+^) group than in the healthy controls (HCs) and the chronic HIV-1 infection (CHI) plus RPR^+^ (CHI&RPR^+^) group. The frequencies of Foxp3^+^CD25^+^CD45RA^+^ and Foxp3^+^Helios^+^CD45RA^+^ Tregs were significantly higher in the RPR^+^, CHI, and CHI&RPR^+^ groups than in HCs, whereas the frequency of CD45RA^+^ Tregs was lower in the CHI&RPR^+^ group than in CHI group. The frequencies of Foxp3^+^CD25^+^CD45RO^+^ and Foxp3^+^Helios^+^CD45RO^+^ Tregs were lower in the RPR^+^, CHI, and CHI&RPR^+^ groups than in HCs. The frequency of intermediate monocytes was inversely correlated with the frequency of CD45RA^+^ Tregs and positively correlated with the frequency of CD45RO^+^ Tregs. These results demonstrate for the first time that intermediate monocytes control the differentiation of Treg subsets in *Treponema pallidum*/HIV-1 co-infections. These findings provide new insights into an immunological mechanism involving monocytes/Tregs in HIV-infected individuals with syphilis.

## Introduction

The management of syphilis in human immunodeficiency virus type 1 (HIV-1)-infected individuals is a global challenge (1–5). HIV-1 and syphilis caused by *Treponema pallidum (T. pallidum)* are bidirectionally synergistic, and co-infection therefore accelerates disease progression (6, 7). Monocytes and regulatory T cells (Tregs) play key roles in regulating pathological immune responses (8–10). The immunological mechanisms involving monocytes and Tregs in *T. pallidum*/HIV-1 co-infection remain incompletely characterized.

Men who have sex with men (MSM) have recently been reported to account for 80% of primary and secondary syphilis diagnoses in the United States, and almost half of these diagnoses were found to be co-infected with HIV-1 (2, 11, 12). Similar findings have been reported in China, where syphilis acquisition among HIV-1-infected MSM has a prevalence between 2.7 and 18.9% (13–18). In 2012, in the United States, there was the early introduction of pre-exposure prophylaxis (PrEP) against HIV as well as the dissemination of evidence that HIV viral suppression may virtually eliminate forward HIV transmission in MSM (19). However, these factors could have led to an increase in syphilis infections or other sexually transmitted diseases such as hepatitis C virus (HCV) occurring after unprotected sex among MSM due to sexual risk compensation (11, 20–22). However, in China, clinical trials for PrEP are just beginning. In addition, HIV-1 and *T. pallidum* act synergistically, increasing the effects of the disease by a factor of eight for HIV and a factor of 5.6–11.4 for syphilis (23, 24). Syphilis increases the recruitment of HIV-1 susceptible inflammatory cells, such as activated macrophages and CD4^+^ and CD8^+^ T cells, to the infection site (25), leading to an increase in HIV viral load, a decrease in CD4^+^ T-cell counts, and high TNF-α and IL-10 levels (26). Patients with HIV-1 infection have a particularly high risk of neurosyphilis (27).

Monocytes are derived from specific hematopoietic precursors in the bone marrow, and they differentiate further into macrophages and dendritic cells (DCs) (8). In 2010, three classifications of monocyte subsets were defined as follows: classical (CD14^++^CD16^−^), intermediate (CD14^++^CD16^+^), and non-classical (CD14^+^CD16^++^) (28). We have previously shown that the frequency of intermediate monocytes and the expression of the surface marker HLA-DR on this subset are higher in individuals with acute or chronic HIV-1 infection than in healthy individuals (29). Monocytes are the cells that respond to the lipoprotein of *T. pallidum*, the causal agent of syphilis (30). The proportion of activated monocytes among peripheral blood mononuclear cells (PBMCs) is significantly higher in patients with neurosyphilis than in those with uncomplicated syphilis (27). However, immunological changes in the three monocyte subsets have not been investigated in HIV-1-infected individuals with syphilis.

Tregs are a critical subset of CD4^+^ T cells involved in the control of immune tolerance through the regulation of immune homeostasis and the limitation of immune activation (9). The nuclear transcription factor Foxp3 has been identified as a specific marker for these cells (31), which also express CD25. Khaitan et al. recently found that, compared with results when considering Foxp3^+^Helios^+^ cells to be Tregs, the traditional combination of Treg specific markers (CD25 and Foxp3) underestimates the proportion of Tregs (32), suggesting that the combination of Foxp3 and Helios may be more reliable in this context. In addition, human Tregs also have highly heterogeneous effects during HIV-1 infection. They may be beneficial, through their suppression of general T-cell activation, or detrimental, due to a weakening of HIV-1-specific responses and the contribution of these cells to viral persistence (9, 10). Human Tregs have been classified into different subsets according to their activation state, with CD45RA^+^ cells considered to constitute the naïve Treg subset and CD45RO^+^ cells defined as the effector/memory Treg subset (33). The absolute number of naïve Tregs has been shown to decrease during acute HIV-1 infection (AHI), whereas effector Treg levels decrease during AHI, with this decrease persisting during chronic HIV-1 infection (CHI) as well (34). While the immunological changes among Tregs in HIV-1 have been investigated intensively, the issue remains unresolved. It was reported that *T. pallidum* can modulate Treg differentiation and activity through TGF-β (35). Patients with syphilis, particularly those with neurosyphilis, have higher percentages of Tregs (36). However, few data are available concerning the changes in Foxp3^+^Helios^+^ Tregs in *T. pallidum*/HIV-1 co-infections.

Intermediate monocytes have been reported to control Helios^+^ Tregs through IL-12, whereas classical monocytes control expansion of the Helios^−^ Treg population through TNF-α during immune thrombocytopenia (37). We have previously shown that intermediate monocyte levels were positively related to the frequency of IFN-γ and IL-4 producing CD4^+^ T helper cells in HIV-1-infected patients (29). We have also demonstrated that *T. pallidum*/HIV-1 co-infection affected the differentiation and function of γδ T cells (38). In this study, we investigated the immunological changes in the levels of monocytes and Tregs and the associations between these cell types in HIV-1-infected MSM with syphilis.

## Methods

### Study Participants

We enrolled 81 participants in this study. These individuals were assigned to four groups on the basis of serologic testing and HIV-1 results, namely, the rapid plasma reagin (RPR^+^) group, the CHI group, the CHI&RPR^+^ group, and the healthy control group.

HIV-1 infection status was determined with the HIV-1/2 antigen/antibody combo enzyme immunoassay (Beijing Wantai Biological Medical Company, Beijing, China). Positive results were confirmed by western blotting for HIV-1/2 (HIV Blot 2.0 MP Diagnostics, Singapore). Participants who had been infected for more than 180 days were considered to have CHI. The diagnosis of syphilis was based on a compatible history and the results of rapid plasma regain (RPR) tests (Shanghai Kehua Company, China) and *T. pallidum* particle agglutination assays (TPPA) (Fujirebio Diagnostics, Inc., Japan). Patients with a latent syphilis infection are defined as those lacking clinical manifestations with the pathogen detected by serologic testing who acquired the infection within the preceding year (39). Twenty-five participants who had been diagnosed with HIV-1 infection for at least 1.5 years (median: 2.1 years, range: 1.5–2.8 years) before enrollment comprised the CHI group. None of these individuals was on ART, and all had high viral loads, low CD4^+^ T-cell counts, and no opportunistic infections or tumors. Seventeen individuals were seropositive in both the TPPA and RPR tests and were without symptoms; and of these individuals who had been infected for <1 year (median: 9 months, range: 5–11 months) before enrollment were defined as the RPR^+^ group. Sixteen participants with HIV-1 and latent syphilis co-infection comprised the CHI&RPR^+^ group, because they met the inclusion criteria of both the CHI and RPR^+^ groups. Seven individuals in the RPR^+^ group and 11 individuals in the CHI&RPR^+^ group had RPR titers ≥1:8 (30). Five individuals in the RPR^+^ group and seven individuals in the CHI&RPR^+^ group received benzathine treatment for syphilis before their enrollment in this study. Twenty-three HIV-negative MSM with negative results in both the TPPA and RPR tests were included in the study as healthy controls. None of the study participants was co-infected with hepatitis B virus/hepatitis C virus, had any other comorbid condition, opportunistic infections, tuberculosis, or autoimmune diseases, and or was a drug user. All of groups were matched for age. The characteristics of the subjects are presented in Table 1.

**Table 1:** Characteristics of the participants enrolled in this study.

	**Healthy controls**	**RPR^**+**^ participants**	**Participants with chronic HIV-1 infection**	**RPR^**+**^ participants with chronic HIV-1 infection**
Cases	23	17	25	16
Ages (years)	34.39 ± 9.41	33.82 ± 6.58	35.80 ± 11.21	31.75 ± 6.11
HIV-RNA (copies/ml)	NA	NA	116157 ± 237206	130778 ± 220541
CD4 (cells/μl)	896 ± 282	851 ± 289	415 ± 211	371 ± 294
RPR titer ≥1:8	NA	7	NA	11

*Data are presented as the means ± SD. NA, not available; RPR, rapid plasma reagin*.

### Cell Surface and Intracellular Cytokine Staining

Cell surface and intracellular cytokine staining were performed as previously described (29). Monocyte phenotypes were analyzed by staining with anti-CD14-FITC (eBioscience), anti-CD16-PE (eBioscience), and anti-HLA-DR-eFluor® 450 (eBioscience) antibodies. For the intracellular staining of Foxp3^+^Helios^+^ Treg cells, we initially stained the cells for surface markers using the following antibodies: anti-CD4-PerCP-cyanine 5.5 (eBioscience), anti-human CD45RO-APC-eFluor® 780 (eBioscience), anti-human CD45RA-eFluor® 450 (eBioscience), and anti-human CD25-PE-Cy7 (eBioscience Inc., San Diego, CA). Cells were then fixed and permeabilized with the Foxp3 transcription factor staining buffer set, according to the manufacturer's instructions (eBioscience Inc., San Diego, CA), and the cells were then incubated with anti-Foxp3-PE (eBioscience) and anti-Helios APC (eBioscience Inc. San Diego CA) antibodies.

### CD4^+^ T-Cell Count and Viral Load

Routine blood CD4^+^ T-cell counts (cells/μl) were measured by four-color flow cytometry with human monoclonal anti-CD4-APC, anti-CD3-FITC, anti-CD8-PE and anti-CD45-PerCP antibodies (BD Multitest^TM^, catalog No. 340499) on peripheral whole-blood samples from each patient according to the manufacturer's instructions. The cells were analyzed on a BD FACS Canto^TM^ II flow cytometry system (BD Biosciences, San Jose, CA).

HIV-1 viral load was determined with an automated real-time PCR-based *m*2000 system (Abbott Molecular Inc., Des Plaines, IL) in accordance with the manufacturer's instructions with a limit of detection of 40 copies/ml.

### Statistical Analysis

Data are expressed as the mean ± standard deviation (SD). We used ANOVA, the Student's *t*-test, or non-parametric tests for statistical analyses. All the reported *p*-values are for two-tailed tests, with the results considered significant if *p* < 0.05. Spearman's rank correlation analyses were performed to assess relationships between two variables. Correlation matrices were displayed as schematic correlograms (40). Statistical analysis was performed with GraphPad Prism software version 5.03 (GraphPad Software, San Diego, California, USA).

## Results

### Characteristics of the Participants

The following four groups of participants were enrolled in this study: 17 RPR^+^, 25 CHI, 16 CHI&RPR^+^, and 23 HC participants. Data on participants' age, viral load, CD4^+^ T-cell counts, and RPR titer are presented in Table 1. The four groups of participants were matched for age. The mean of CD4^+^ T-cell counts in the HC and RPR^+^ groups were higher than those in the CHI and CHI&RPR^+^ groups (*p* < 0.01). RPR titer did not differ significantly between the RPR^+^ and CHI& RPR^+^ groups.

### Alterations of the Three Monocyte Subsets and Their HLA-DR Expression During *T. pallidum*/HIV-1 Co-infection

**Figure 1:**
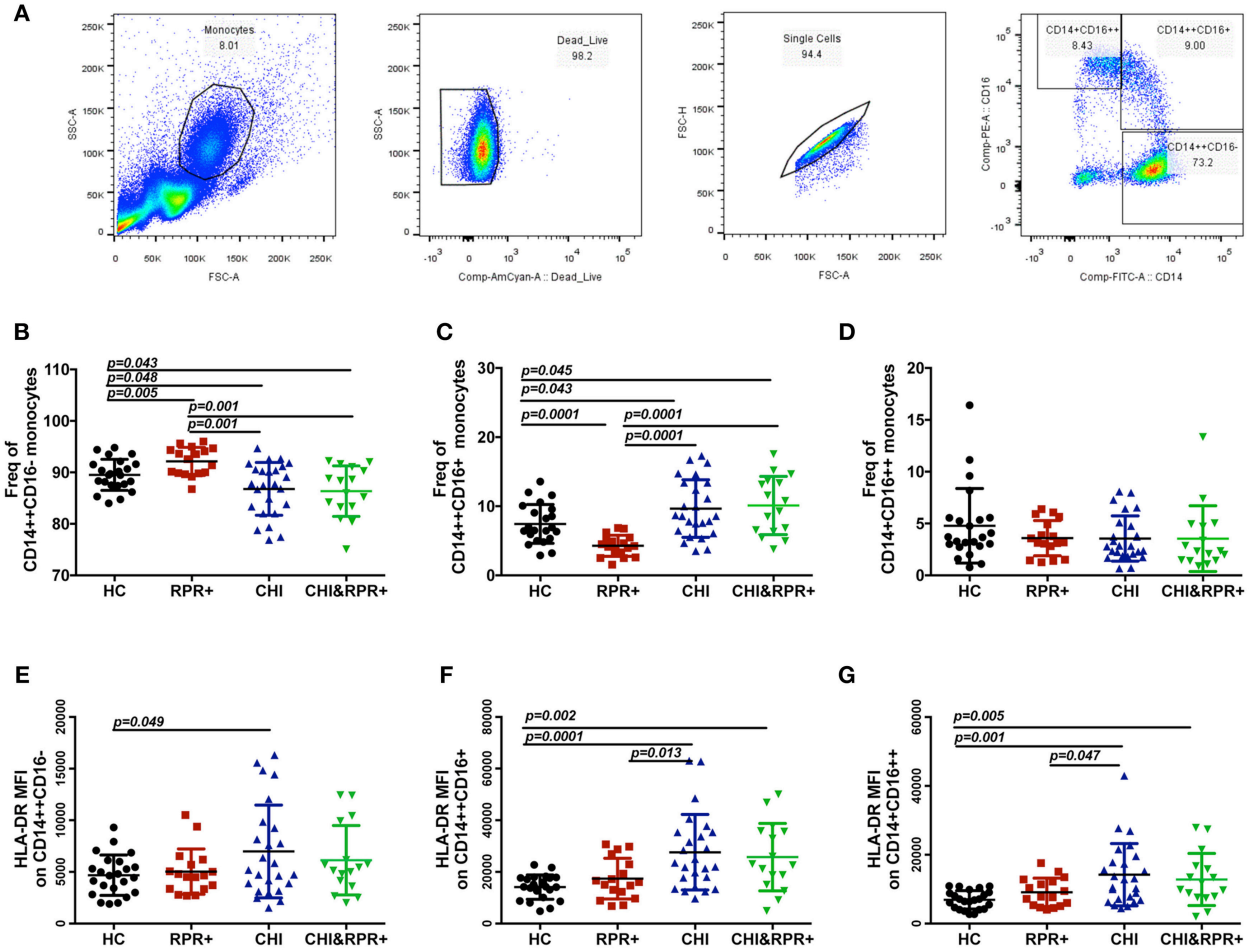
The alterations of the three monocyte subsets and their HLA-DR expression during *T. pallidum*/HIV-1 co-infection. **(A)** The gating strategy used for analysis of the classical CD14^++^CD16^−^, intermediate CD14^++^CD16^+^, and non-classical CD14^+^CD16^++^ monocyte subsets is indicated. The frequencies of CD14^++^CD16^−^ **(B)**, CD14^++^CD16^+^ **(C)**, and CD14^+^CD16^++^ **(D)** monocytes and the MFI values for surface HLA-DR expression on the CD14^++^CD16^−^ **(E)**, CD14^++^CD16^+^ **(F)**, and CD14^+^CD16^++^ **(G)** monocytes were analyzed by flow cytometry in healthy controls (HCs), patients with syphilis infection (RPR^+^), patients with chronic HIV-1 infection (CHI), and patients with *T. pallidum*/HIV-1 co-infection (CHI&RPR^+^). Significance was assessed by calculating *p-*values using ANOVA, Student's *t*-tests, or Mann–Whitney *U-*tests, with values of *p* < 0.05 considered significant.

The gating strategy used for monocyte subsets is shown in Figure 1A. The frequency of classical monocytes was higher in the RPR^+^ group and lower in the CHI group than in the HCs, and was lower in the CHI&RPR^+^ group than in the RPR^+^ group (Figure 1B). The frequency of intermediate monocytes was lower in the RPR^+^ group and higher in the CHI group than in the HCs, and the frequency of intermediate monocytes in the CHI&RPR^+^ group was higher than that in the RPR^+^ group (Figure 1C). Regarding the non-classical monocytes, there were no differences among the four groups (Figure 1D). Furthermore, we characterized the altered expression of HLA-DR on the three monocyte subsets. On classical monocytes, the expression of the activation marker HLA-DR was higher in the CHI groups than in the HCs (Figure 1E), but on the intermediate and non-classical monocyte subsets, this marker was significantly higher in the CHI and CHI&RPR^+^ groups than in the HC and RPR^+^ groups (all *p* < 0.05, Figures 1F,G).

### Immunological Changes in Foxp3^+^Helios^+^ and Foxp3^+^CD25^+^ Tregs During *T. pallidum*/HIV-1 Co-infection

**Figure 2:**
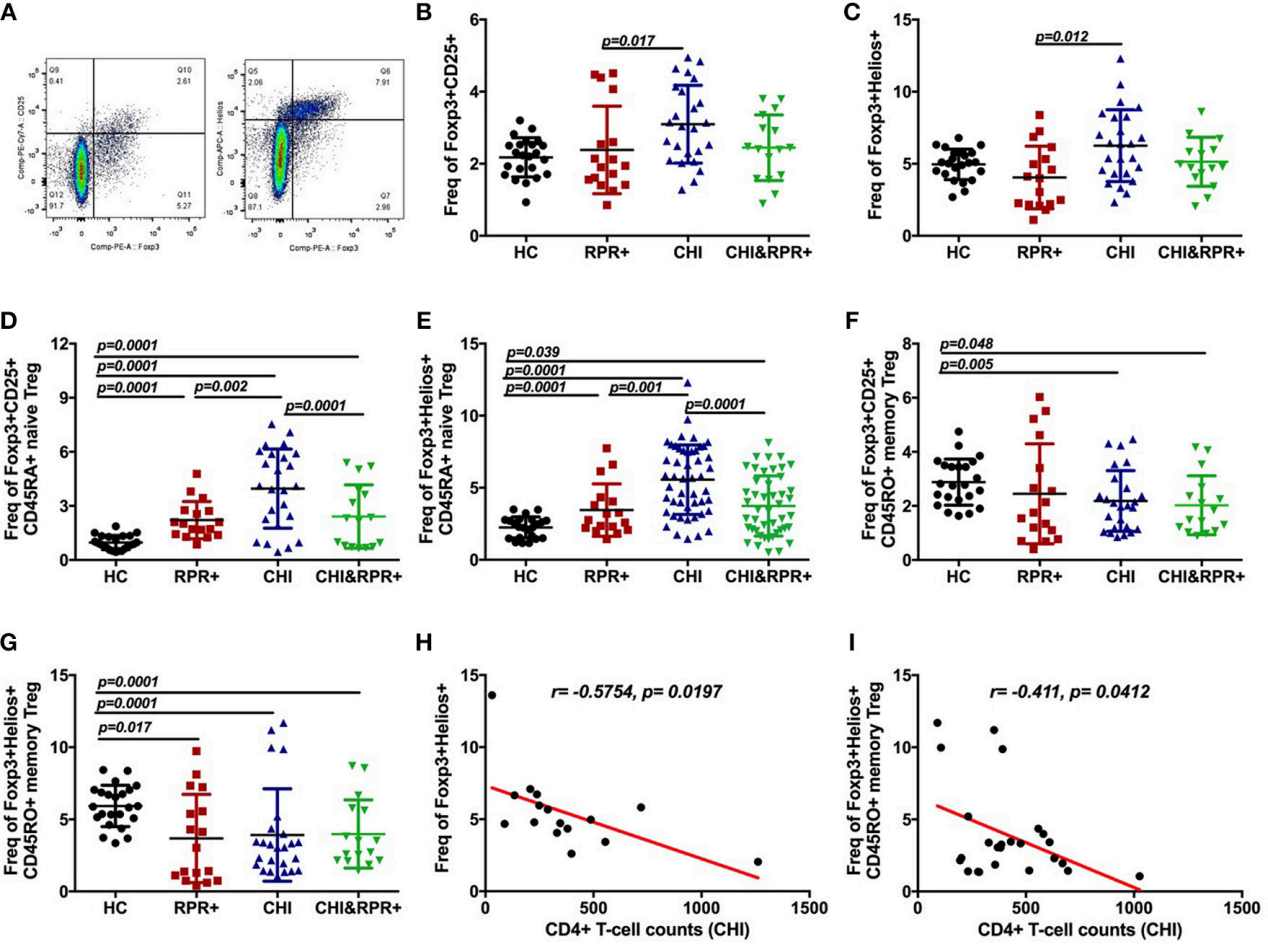
Immunological changes in Foxp3^+^Helios^+^ and Foxp3^+^CD25^+^ Tregs during *T. pallidum*/HIV-1 co-infection. The gating strategy for the analysis of Foxp3^+^CD25^+^ and Foxp3^+^Helios^+^ **(A)** cells is indicated. The frequencies of Foxp3^+^CD25^+^ Treg cells **(B)** and Foxp3^+^Helios^+^ Treg cells **(C)** were analyzed by flow cytometry in the HC, RPR^+^, CHI, and CHI&RPR^+^ groups. Frequencies of Foxp3^+^CD25^+^CD45RA^+^ Treg cells **(D)**, Foxp3^+^Helios^+^CD45RA^+^ Treg cells **(E)**, Foxp3^+^CD25^+^CD45RO^+^ Tregs **(F)**, and Foxp3^+^Helios^+^CD45RO^+^ Tregs **(G)** were analyzed by flow cytometry in the HC, RPR^+^, CHI, and CHI&RPR^+^ groups. The correlations between the CD4^+^ T-cell counts and the frequencies of Foxp3^+^Helios^+^ Treg cells **(H)** and Foxp3^+^Helios^+^CD45RO^+^ cells **(I)** in the CHI group were evaluated with Spearman's rank correlation test. Significance was assessed by calculating *p-*values using ANOVA, Student's *t*-tests, or Mann–Whitney *U-*tests, with values of *p* < 0.05 considered significant.

The strategy for gating Foxp3^+^Helios^+^ Tregs and Foxp3^+^CD25^+^ Tregs from CD4^+^ T cells is presented in Figure 2A. We found that the frequency of Foxp3^+^Helios^+^ Tregs was higher than that of Foxp3^+^CD25^+^ Tregs in each group as shown in Figures 2B–G. The frequencies of Foxp3^+^CD25^+^ and Foxp3^+^Helios^+^ Tregs were significantly higher in the CHI groups than in the RPR^+^ group (Figures 2B,C). The frequency of Foxp3^+^Helios^+^ Tregs was inversely correlated with CD4^+^ T-cell counts in the CHI group (Figure 2H).

We further separated Tregs into Foxp3^+^CD45RA^+^ naïve Tregs and Foxp3^+^CD45RO^+^ memory Tregs. The frequency of Foxp3^+^CD25^+^CD45RA^+^ Tregs was higher in the RPR^+^, CHI and CHI&RPR^+^ groups than in HCs and was higher in the CHI group than in the CHI&RPR^+^ group (Figure 2D). Similar results were also observed regarding the frequencies of Foxp3^+^Helios^+^CD45RA^+^ Tregs in the four groups (Figure 2E). In addition, the frequency of Foxp3^+^CD25^+^CD45RO^+^ Tregs was significantly lower in the CHI&RPR^+^ and CHI groups than in HCs (Figure 2F), and similar lower frequencies were also observed for Foxp3^+^Helios^+^CD45RO^+^ Tregs within the RPR^+^, CHI, and CHI&RPR^+^ groups than in HCs (Figure 2G). The frequency of Foxp3^+^Helios^+^CD45RO^+^ Treg cells was inversely correlated with CD4^+^ T-cell counts in the CHI group (Figure 2I).

### Correlation Between Monocyte Subsets and Foxp3+ Tregs During *T. pallidum*/HIV-1 Co-infection

**Figure 3:**
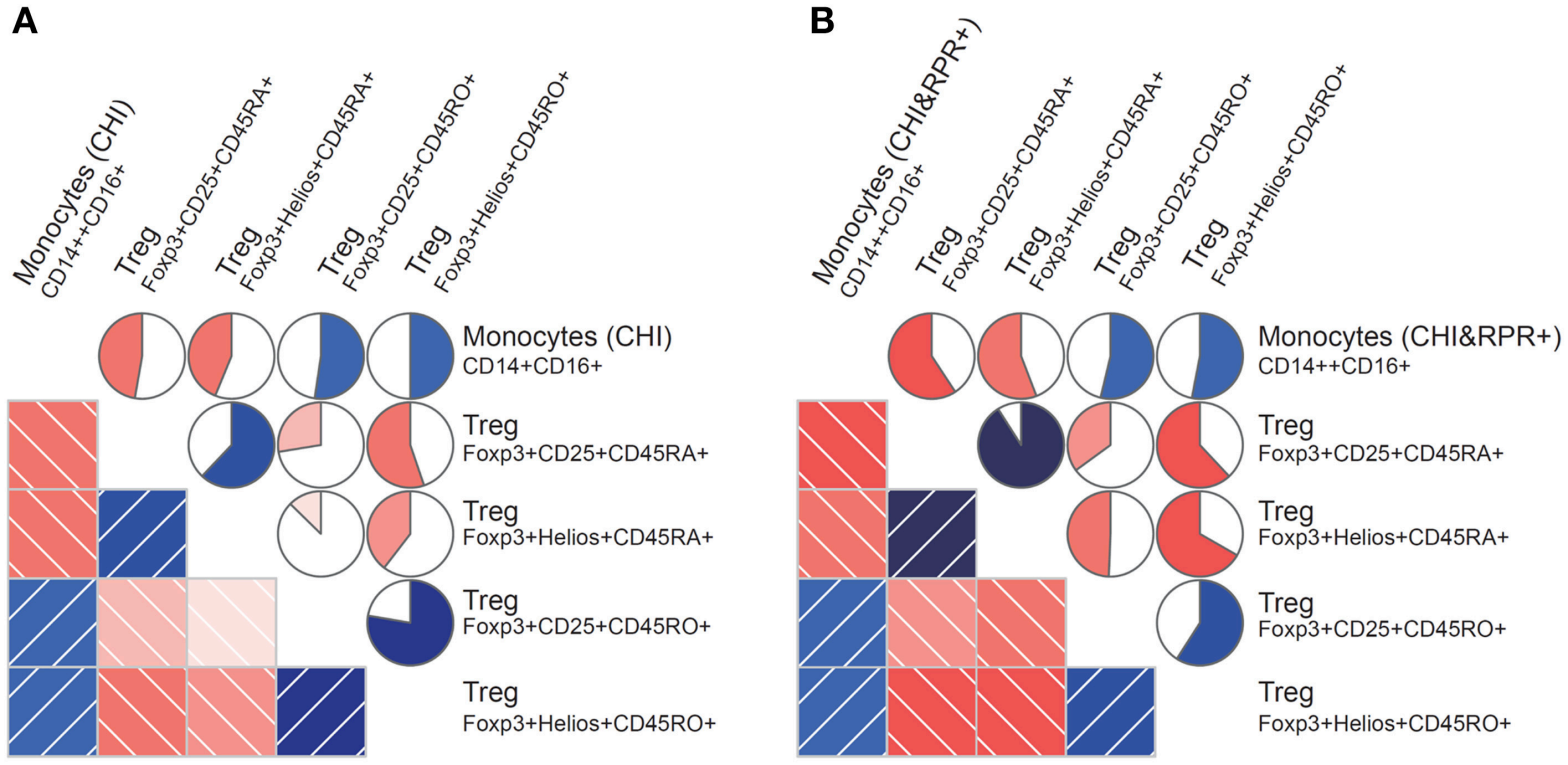
Correlations between monocyte subsets and Foxp3^+^ Tregs during *T. pallidum*/HIV-1 co-infection. Monocyte subset frequencies were correlated with Foxp3^+^ Treg cell frequencies from patients. **(A)** The subset of CD14^++^CD16^+^ monocytes (CHI) correlated positively with the Foxp3^+^CD25^+^CD45RO^+^ and Foxp3^+^Helios^+^CD45RO^+^ Treg subsets and negatively with the Foxp3^+^CD25^+^CD45RA^+^ and Foxp3^+^Helios^+^CD45RA^+^ subsets; **(B)** The subset of CD14^++^CD16^+^ monocytes (CHI&RPR+) also correlated positively with the Foxp3^+^CD25^+^CD45RO^+^ and Foxp3^+^Helios^+^CD45RO^+^ Treg subsets and negatively with the Foxp3^+^CD25^+^CD45RA^+^ and Foxp3^+^Helios^+^CD45RA^+^ subsets. The blue and red colors represent positive and negative correlations, respectively, between the CD14^++^CD16^+^ monocyte subset levels and Foxp3^+^ Treg cell levels in the CHI and CHI&RPR^+^ groups. The darker and more saturated the color, the greater the magnitude of the correlation. Correlation matrices were displayed as schematic correlograms (40).

We found that the frequency of intermediate monocytes was inversely correlated with the frequency of Foxp3^+^CD25^+^CD45RA^+^ Tregs and Foxp3^+^Helios^+^CD45RA^+^ Tregs in the CHI group (Figure 3A) and the CHI&RPR^+^ group (Figure 3B). In contrast to the frequency of CD45RA^+^ Tregs, the frequency of intermediate monocytes was positively correlated with the frequency of Foxp3^+^CD25^+^CD45RO^+^ Tregs and Foxp3^+^Helios^+^CD45RO^+^ Tregs in the CHI group (Figure 3A) and the CHI&RPR^+^ group (Figure 3B). These data suggest that intermediate monocytes control the differentiation of Treg subsets during HIV-1 infection alone and in combination with *T. pallidum* co-infection.

## Discussion

In this study, we evaluated the phenotypic and immunological changes in three monocyte subsets and Tregs and explored the associations between these cell types during *T. pallidum*/HIV-1 co-infection. We found that the frequency of classical monocytes increased during syphilis infection. We also found that the naïve Treg population expanded during HIV-1 or syphilis infection alone and during *T. pallidum*/HIV-1 co-infection, whereas the memory Treg population decreased in size. The frequency of naïve Tregs was lower during HIV-1 infection than during *T. pallidum*/HIV-1 co-infection but was still higher than that observed during syphilis infection. Furthermore, during HIV-1 infection and *T. pallidum*/HIV-1 co-infection, the frequency of intermediate monocytes was inversely correlated with the frequency of naïve Tregs but positively correlated with the frequency of memory Tregs.

The levels of monocytes and Tregs and their associations changed during *T. pallidum*/HIV-1 co-infection. Classical monocytes clear pathogens by phagocytosis in the early stages of syphilis. The expansion of the naïve Treg population and the decrease in the memory Treg population may reflect the hyperactivation, inflammatory response, and dominant cell depletion observed in HIV-1 and syphilis infections separately and in *T. pallidum*/HIV-1 co-infection. The three monocyte subsets were found to have different effects on Treg-cell differentiation in HIV-1 infection and *T. pallidum*/HIV-1 co-infection. Clarification of the ways in which monocyte subsets influence adaptive responses may therefore make it possible to boost or prevent pathological effects during HIV-1 infection and *T. pallidum*/HIV-1 co-infection.

Monocytes have many immunological functions linked to the innate and adaptive immune systems. Classical monocytes are mostly involved in phagocytosis to prevent pathogen invasion, whereas intermediate monocytes are principally involved in antigen presentation and inflammatory responses, and non-classical monocytes are mostly involved in immune surveillance (41). *T. pallidum* contains abundant lipoproteins, which can activate macrophages and DCs through Toll-like receptor (TLR) 2-dependent signaling pathways (3). Although *T. pallidum* expresses abundant lipoproteins, these molecules reside predominantly below the surface of this pathogen (3). As a result, these molecules are not readily accessible to TLRs or other pattern recognition receptors (PRRs) present on monocytes/macrophages or DCs (30). Classical monocytes play an important role in the progression of the immune response. They are recruited within the first few hours of infection. On arrival, they exert an immediate and potent immune response by producing high levels of pro-inflammatory cytokines and IL-18 to activate NK cells (42). We found that, contrary to what is observed during HIV-1 infection, the frequency of classical monocytes increased during the early stages of syphilis infection, with the host immune system defending the host against *T. pallidum* through phagocytosis and cell-mediated immune responses (35).

Tregs originate from the thymus and differentiate into naïve Tregs in the peripheral blood. Following stimulation with an external antigen, naïve Tregs further differentiate into memory Tregs. Tregs can inhibit the immune response, allowing HIV-1 (10) and *T. pallidum* (35) to escape host immune defenses, resulting in the development of chronic disease. In syphilis patients, Tregs are induced and regulated by the secretion of cytokines elicited by *T. pallidum* and other pathways (35). Treg percentages and suppressive functions in the peripheral blood have been shown to be significantly higher in syphilis patients than in HCs at an early stage of infection (36). The Foxp3^+^Helios^+^CD45RA^+^ and Foxp3^+^CD25^+^CD45RA^+^ Treg populations expanded during syphilis and during *T. pallidum*/HIV-1 co-infection. This expansion may reflect enhanced Treg generation in the thymus (43) or increases in Treg survival (10). The frequencies of Foxp3^+^Helios^+^CD45RA^+^ and Foxp3^+^CD25^+^CD45RA^+^ Tregs were lower during *T. pallidum*/HIV-1 co-infection than during infection with HIV-1 alone. The resulting lower level of immune suppression may contribute to inappropriate or exaggerated immune activation, leading to immune-mediated tissue damage. Greater expansion of the naïve Treg population is associated with disease progression (44). Significant decreases in the absolute numbers of Foxp3^+^CD25^+^CD45RO^+^ Treg cells have been reported in patients with acute and chronic HIV infection (34). Foxp3^+^Helios^+^CD45RO^+^ Treg cells and Foxp3^+^CD25^+^CD45RO^+^ Treg cells were depleted relative to HCs in patients with HIV-1 infection and/or syphilis. Memory Treg depletion appears to occur early in HIV-1 infection and persists during the chronic phase of the infection, presumably reflecting a preferential loss of the memory Treg subset (9). An impairment of memory Treg homeostasis has been reported early in HIV-1 infection (34). The lower frequency of memory Tregs during HIV-1 infection and/or syphilis indicates a lower level of specific T-cell inhibition, which may be beneficial in the fight against pathogens.

DCs are derived from monocytes and are professional antigen-presenting cells (APCs) that are more likely to induce Tregs in HIV-1-infected individuals than in HCs (45). DCs activate T cells by integrating multiple signals, including antigen, costimulatory molecule, cytokines, and chemokines (46). Nguyen et al. found that serum amyloid A induced regulatory T cells through monocyte activation by mitogen signals, with IL-1β and IL-6 directly involved in the serum amyloid A (SAA)-mediated proliferation of Tregs (47). In syphilis patients, TpF1 promotes the release of IL-10 and TGF-β from monocytes, potentially driving Treg cell differentiation (35). In the CHI and CHI&RPR^+^ groups in our study, the frequency of intermediate monocytes was inversely correlated with the frequency of CD45RA^+^ Tregs but was positively correlated with the frequency of CD45RO^+^ Tregs. This finding suggests that intermediate monocytes control the differentiation of Treg subsets during HIV-1 infection and *T. pallidum*/HIV-1 co-infection.

One limitation of the study is that we only evaluated the association between monocyte subsets and Foxp3^+^ Tregs during *T. pallidum*/HIV-1 co-infection by phenotyping. It would be better to use fresh samples from the same individuals to perform monocyte and Treg co-culture experiments, which could provide clues regarding possible mechanisms. However, according to the “Chinese Guidelines for the Use of Antiretroviral Agents in HIV-1-Infected Adults and Adolescents (Version 2018)” (48), HIV-1-infected individuals should be treated immediately once confirmed, and the viral loads of most of the HIV-1-infected patients will become undetectable. Currently, it is not very easy to obtain enough fresh samples from patients co-infected with *T. pallidum*/HIV-1 (with high viral loads). However, it will be worthwhile for further studies to investigate the mechanisms of immunological changes in monocyte subsets and their associations with Foxp3^+^ regulatory T cells in *T. pallidum*/HIV-1-infected individuals, and a larger number of these individuals will be required to do so.

Taken together, our results for the first time explore the immunological changes in monocyte and Treg subsets in HIV-infected individuals with syphilis. We also analyzed the association between intermediate monocytes and Treg subsets. Our findings shed new light on the immunological mechanisms underlying *T. pallidum*/HIV-1 co-infection, which will provide important information for the development of effective prevention and intervention strategies.

## Ethics Statement

All the participants came from the Beijing PRIMO Clinical Cohort, which ran from 2007 to 2012. They provided written informed consent for participation in the study and for the storage and use of their clinical samples for research. This study and other related experiments were approved by the Beijing Youan Hospital Research Ethics Committee, and informed consent was obtained in accordance with the Declaration of Helsinki. The study was carried out in accordance with approved guidelines and regulations.

## Author Contributions

BS, LfL, TZ, and HW conceived the study, designed the experiments, and analyzed the data. NG, XY, TS, LL, and GL performed the experiments. LfL, TJ, YG, TZ, BS, and HW contributed to reagents and materials. NG, LfL, and BS wrote the article. All authors read and approved the final manuscript.

### Conflict of Interest Statement

The authors declare that the research was conducted in the absence of any commercial or financial relationships that could be construed as a potential conflict of interest.

## References

[1] Novak RM Ghanem A Hart R Ward D Armon C Buchacz K. Risk factors and incidence of syphilis in human immunodeficiency virus (HIV)-infected persons: the HIV outpatient study, 1999-2015. Clin Infect Dis. (2018) 67:1750–9. 10.1093/cid/ciy348 29688270 PMC11307151

[2] de Voux A Bernstein KT Bradley H Kirkcaldy RD Tie Y Shouse RL Syphilis testing among sexually active men who have sex with men and who are receiving medical care for human immunodeficiency virus in the United States: medical monitoring project, 2013-2014. Clin Infect Dis. (2019) 68:934–9. 10.1093/cid/ciy571 29985985 PMC6563935

[3] Peeling RW Mabey D Kamb ML Chen XS Radolf JD Benzaken AS. Syphilis. Nat Rev Dis Primers. (2017) 3:17073. 10.1038/nrdp.2017.73 29022569 PMC5809176

[4] Hook ER. Syphilis. Lancet. (2017) 389:1550–7. 10.1016/S0140-6736(16)32411-4 27993382

[5] Roberts CP Klausner JD. Global challenges in human immunodeficiency virus and syphilis coinfection among men who have sex with men. Exp. Rev Anti Infect Ther. (2016) 14:1037–46. 10.1080/14787210.2016.1236683 27626361 PMC5859941

[6] Pathela P Braunstein SL Blank S Shepard C Schillinger JA. The high risk of an HIV diagnosis following a diagnosis of syphilis: a population-level analysis of New York City men. Clin Infect Dis. (2015) 61:281–7. 10.1093/cid/civ289 25870333

[7] Solomon MM Mayer KH Glidden DV Liu AY McMahan VM Guanira JV. Syphilis predicts HIV incidence among men and transgender women who have sex with men in a preexposure prophylaxis trial. Clin Infect Dis. (2014) 59:1020–6. 10.1093/cid/ciu450 24928295 PMC4166980

[8] Wacleche VS Tremblay CL Routy JP Ancuta P. The biology of monocytes and dendritic cells: contribution to HIV pathogenesis. Viruses. (2018) 10:E65. 10.3390/v10020065 29415518 PMC5850372

[9] Simonetta F Bourgeois C. CD4+FOXP3+ regulatory T-cell subsets in human immunodeficiency virus infection. Front Immunol. (2013) 4:215. 10.3389/fimmu.2013.00215 23908654 PMC3727053

[10] Chevalier MF Weiss L. The split personality of regulatory T cells in HIV infection. Blood. (2013) 121:29–37. 10.1182/blood-2012-07-409755 23043072

[11] Schillinger JA Slutsker JS Pathela P Klingler EJ Hennessy RR Toro B. The epidemiology of syphilis in New York City: historic trends and the current outbreak among men who have sex with men, 2016. Sex Transm Dis. (2018) 45:S48–54. 10.1097/OLQ.0000000000000796 29465651

[12] Refugio ON Klausner JD. Syphilis incidence in men who have sex with men with human immunodeficiency virus comorbidity and the importance of integrating sexually transmitted infection prevention into HIV care. Expert Rev Anti Infect Ther. (2018) 16:321–31. 10.1080/14787210.2018.1446828 29489420 PMC6352966

[13] Yang T Chen Q Li D Wang T Gou Y Wei B. High prevalence of syphilis, HBV, and HCV co-infection, and low rate of effective vaccination against hepatitis B in HIV-infected patients in West China hospital. J Med Virol. (2018) 90:101–8. 10.1002/jmv.24912 28792076

[14] Wong NS Chen L Tucker JD Zhao P Goh BT Poon CM. Distribution of reported syphilis cases in South China: spatiotemporal analysis. Sci Rep. (2018) 8:9090. 10.1038/s41598-018-27173-y 29904141 PMC6002518

[15] Chen L Mahapatra T Fu G Huang S Zheng H Tucker JD. Male clients of male sex workers in China: an ignored high-risk population. J Acquir Immune Defic Syndr. (2016) 71:316–22. 10.1097/QAI.0000000000000833 26871882 PMC4755356

[16] Jia Z Huang X Wu H Zhang T Li N Ding P. HIV burden in men who have sex with men: a prospective cohort study 2007-2012. Sci Rep. (2015) 5:11205. 10.1038/srep11205 26135810 PMC5393284

[17] Wu Z Xu J Liu E Mao Y Xiao Y Sun X. HIV and syphilis prevalence among men who have sex with men: a cross-sectional survey of 61 cities in China. Clin Infect Dis. (2013) 57:298–309. 10.1093/cid/cit210 23580732 PMC3689345

[18] Guo H Wei JF Yang H Huan X Tsui SK Zhang C. Rapidly increasing prevalence of HIV and syphilis and HIV-1 subtype characterization among men who have sex with men in Jiangsu, China. Sex Transm Dis. (2009) 36:120–5. 10.1097/OLQ.0b013e31818d3fa0 19125142

[19] Rodger AJ Cambiano V Bruun T Vernazza P Collins S van Lunzen J. Sexual activity without condoms and risk of HIV transmission in serodifferent couples when the HIV-positive partner is using suppressive antiretroviral therapy. JAMA. (2016) 316:171–81. 10.1001/jama.2016.5148 27404185

[20] Traeger MW Schroeder SE Wright EJ Hellard ME Cornelisse VJ Doyle JS. Effects of pre-exposure prophylaxis for the prevention of human immunodeficiency virus infection on sexual risk behavior in men who have sex with men: a systematic review and meta-analysis. Clin Infect Dis. (2018) 67:676–86. 10.1093/cid/ciy182 29509889

[21] Barreiro P. Hot news: sexually transmitted infections on the rise in PrEP users. Aids Rev. (2018) 20:71. 29628512

[22] Price JC McKinney JE Crouch PC Dillon SM Radix A Stivala A. Sexually acquired hepatitis C infection in HIV-uninfected men who have sex with men using pre-exposure prophylaxis against HIV. J Infect Dis. (2018). 10.1093/infdis/jiy670. [Epub ahead of print]. 30462305

[23] Zhou Y Li D Lu D Ruan Y Qi X Gao G. Prevalence of HIV and syphilis infection among men who have sex with men in China: a meta-analysis. Biomed Res Int. (2014) 2014:620431. 10.1155/2014/620431 24868533 PMC4017804

[24] Xu J Han X Reilly KH Shang H. New features of the HIV epidemic among men who have sex with men in China. Emerg Microbes Infect. (2013) 2:e45. 10.1038/emi.2013.45 26038478 PMC3820985

[25] Radolf JD Deka RK Anand A Smajs D Norgard MV Yang XF. *Treponema pallidum*, the syphilis spirochete: making a living as a stealth pathogen. Nat Rev Microbiol. (2016) 14:744–59. 10.1038/nrmicro.2016.141 27721440 PMC5106329

[26] Knudsen A Benfield T Kofoed K. Cytokine expression during syphilis infection in HIV-1-infected individuals. Sex Transm Dis. (2009) 36:300–4. 10.1097/OLQ.0b013e318193ca26 19265730

[27] Ho EL Maxwell CL Dunaway SB Sahi SK Tantalo LC Lukehart SA Neurosyphilis increases human immunodeficiency virus (HIV)-associated central nervous system inflammation but does not explain cognitive impairment in HIV-infected Individuals with Syphilis. Clin Infect Dis. (2017) 65:943–8. 10.1093/cid/cix473 28525592 PMC5849093

[28] Ziegler-Heitbrock L Ancuta P Crowe S Dalod M Grau V Hart DN. Nomenclature of monocytes and dendritic cells in blood. Blood. (2010) 116:e74–e80. 10.1182/blood-2010-02-258558 20628149

[29] Chen P Su B Zhang T Zhu X Xia W Fu Y. Perturbations of monocyte subsets and their association with T helper cell differentiation in acute and chronic HIV-1-infected patients. Front Immunol. (2017) 8:272. 10.3389/fimmu.2017.00272 28348563 PMC5347116

[30] Cruz AR Ramirez LG Zuluaga AV Pillay A Abreu C Valencia CA. Immune evasion and recognition of the syphilis spirochete in blood and skin of secondary syphilis patients: two immunologically distinct compartments. PLoS Negl Trop Dis. (2012) 6:e1717. 10.1371/journal.pntd.0001717 22816000 PMC3398964

[31] Sakaguchi S Sakaguchi N Asano M Itoh M Toda M. Immunologic self-tolerance maintained by activated T cells expressing IL-2 receptor alpha-chains (CD25). Breakdown of a single mechanism of self-tolerance causes various autoimmune diseases. J Immunol. (1995) 155:1151–64. 7636184

[32] Khaitan A Kravietz A Mwamzuka M Marshed F Ilmet T Said S. FOXP3+Helios+ regulatory T cells, immune activation, and advancing disease in HIV-infected children. J Acquir Immune Defic Syndr. (2016) 72:474–84. 10.1097/QAI.0000000000001000 27003495 PMC4942350

[33] Valverde-Villegas JM Matte MC de Medeiros RM Chies JA. New insights about Treg and Th17 cells in HIV infection and disease progression. J Immunol Res. (2015) 2015:647916. 10.1155/2015/647916 26568963 PMC4629044

[34] Simonetta F Lecuroux C Girault I Goujard C Sinet M Lambotte O. Early and long-lasting alteration of effector CD45RA(-)Foxp3(high) regulatory T-cell homeostasis during HIV infection. J Infect Dis. (2012) 205:1510–9. 10.1093/infdis/jis235 22457280 PMC3989210

[35] Babolin C Amedei A Ozolins D Zilevica A D'Elios MM de Bernard M. TpF1 from *Treponema pallidum* activates inflammasome and promotes the development of regulatory T cells. J Immunol. (2011) 187:1377–84. 10.4049/jimmunol.1100615 21709157

[36] Li K Wang C Lu H Gu X Guan Z Zhou P. Regulatory T cells in peripheral blood and cerebrospinal fluid of syphilis patients with and without neurological involvement. PLoS Negl Trop Dis. (2013) 7:e2528. 10.1371/journal.pntd.0002528 24244772 PMC3820703

[37] Zhong H Yazdanbakhsh K. Differential control of Helios(+/–) Treg development by monocyte subsets through disparate inflammatory cytokines. Blood. (2013) 121:2494–502. 10.1182/blood-2012-11-469122 23365462 PMC3612859

[38] Li Z Lu X Hu Z Luo Z Jiang W Wu H. Syphilis infection differentially regulates the phenotype and function of gammadelta T cells in HIV-1-infected patients depends on the HIV-1 disease stage. Front Immunol. (2017) 8:991. 10.3389/fimmu.2017.00991 28871259 PMC5566620

[39] Workowski KA Bolan GA. Sexually transmitted diseases treatment guidelines, 2015. MMWR Recomm Rep. (2015) 64:1–137. 26042815 PMC5885289

[40] Friendly M Corrgrams: exploratory displays for correlation matrices. Am Stat. (2002) 56:316–24. 10.1198/000313002533

[41] Sprangers S de Vries TJ Everts V. Monocyte heterogeneity: consequences for monocyte-derived immune cells. J Immunol Res. (2016) 2016:1475435. 10.1155/2016/1475435 27478854 PMC4958468

[42] Serti E Werner JM Chattergoon M Cox AL Lohmann V Rehermann B. Monocytes activate natural killer cells via inflammasome-induced interleukin 18 in response to hepatitis C virus replication. Gastroenterology. (2014) 147:209–20. 10.1053/j.gastro.2014.03.046 24685721 PMC4469643

[43] Bandera A Ferrario G Saresella M Marventano I Soria A Zanini F. CD4+ T cell depletion, immune activation and increased production of regulatory T cells in the thymus of HIV-infected individuals. PLOS ONE. (2010) 5:e10788. 10.1371/journal.pone.0010788 20520721 PMC2875388

[44] Xing S Fu J Zhang Z Gao Y Jiao Y Kang F. Increased turnover of FoxP3high regulatory T cells is associated with hyperactivation and disease progression of chronic HIV-1 infection. J Acquir Immune Defic Syndr. (2010) 54:455–62. 10.1097/QAI.0b013e3181e453b9 20585263

[45] Carbonneil C Donkova-Petrini V Aouba A Weiss L. Defective dendritic cell function in HIV-infected patients receiving effective highly active antiretroviral therapy: neutralization of IL-10 production and depletion of CD4+CD25+ T cells restore high levels of HIV-specific CD4+ T cell responses induced by dendritic cells generated in the presence of IFN-alpha. J Immunol. (2004) 172:7832–40. 10.4049/jimmunol.172.12.7832 15187167

[46] Mueller SN Gebhardt T Carbone FR Heath WR. Memory T cell subsets, migration patterns, and tissue residence. Annu Rev Immunol. (2013) 31:137–61. 10.1146/annurev-immunol-032712-095954 23215646

[47] Nguyen KD Macaubas C Truong P Wang N Hou T Yoon T. Serum amyloid A induces mitogenic signals in regulatory T cells via monocyte activation. Mol Immunol. (2014) 59:172–9. 10.1016/j.molimm.2014.02.011 24632292 PMC4068397

[48] AIDS and Hepatitis C Professional Group SOID Prevention CCFD. Chinese guidelines for diagnosis and treatment of HIV/AIDS. Chin J Intern Med. (2018) 57:867–84. 10.3760/cma.j.issn.0578-1426.2018.12.002 30486555

